# Enhanced Recovery Protocol Post‐TBI (ERA‐TBI): Factors Determining Morbidity and Mortality

**DOI:** 10.1155/nri/8974212

**Published:** 2026-07-24

**Authors:** George-Paul O’Byrne, Shreya Sankar, Suhani Sharma, David Beniameen, Amritha Ramachandran, Shah Al Raziq

**Affiliations:** ^1^ Department of Surgery, University Hospital Limerick, St Nessan’s Rd Dooradoyle Co., Limerick V94 F858, Ireland, ul.ie; ^2^ School of Medicine, Royal College of Surgeons in Ireland, 123 St. Stephen’s Green Dublin Co., Dublin DO2 YN77, Ireland, rcsi.ie

## Abstract

Traumatic brain injury (TBI) remains a leading cause of death and long‐term disability worldwide, with substantial heterogeneity in recovery trajectories despite advances in acute neurocritical care. Enhanced Recovery After Surgery (ERAS) programmes, which integrate evidence‐based, multidisciplinary perioperative care bundles, have demonstrated consistent benefits across multiple surgical specialities and emerging promise in neurosurgical populations. However, the application of enhanced recovery principles to the recovery phase following TBI remains fragmented and insufficiently synthesised. This narrative review examines recovery‐phase factors influencing morbidity and mortality after TBI within the conceptual framework of Enhanced Recovery After TBI (ERA‐TBI). Focussing specifically on postacute recovery, we synthesise evidence across three interrelated domains: rehabilitation strategies, patient demographics and preexisting conditions. Perioperative surgical techniques and acute intensive care management are considered only insofar as they contextualise recovery‐phase outcomes. The reviewed literature demonstrates that early, intensive and multidisciplinary rehabilitation, particularly early mobilisation and structured cognitive or neuropsychological interventions, is associated with improved neurological recovery, functional independence, quality of life and reduced hospital length of stay, without evidence of increased mortality. Emerging technologies, including virtual reality and telerehabilitation, show promising cognitive and functional benefits, although evidence remains limited by heterogeneity and small sample sizes. Patient‐level factors strongly modify recovery potential: advanced age, frailty and socioeconomic disadvantage consistently predict poorer outcomes, while access to high‐intensity inpatient rehabilitation is a key mediator of morbidity. Preexisting metabolic, cardiovascular and neuropsychiatric comorbidities further exacerbate secondary brain injury, prolong hospitalisation and increase postdischarge mortality risk. Overall, ERA‐TBI pathways must move beyond uniform care bundles towards stratified, patient‐centred recovery models that integrate biological reserve, social context and comorbidity burden. Standardising the recovery phase through an ERA‐TBI framework offers a critical opportunity to reduce variability in outcomes, optimise functional recovery and address the growing global burden of TBI‐related disability.

## 1. Introduction

Traumatic brain injury (TBI) is a major cause of death and disability worldwide. The Global Burden of Disease (GBD) 2021 analysis estimated approximately 20.84 million new TBI cases in 2021, with a global age‐standardised incidence of 259 per 100,000 [1]. In the United States, the CDC reported 69,473 TBI‐related deaths in 2021, equivalent to about 190 per day and 214,110 TBI‐related hospitalisations in 2020, excluding cases managed only in emergency or primary care or untreated altogether [2]. These figures highlight the continuing global and national burden of TBI.

Enhanced Recovery After Surgery (ERAS) programmes apply evidence‐based, perioperative care bundles to improve outcomes across surgical specialities. In neurosurgery, ERAS has been adapted to span the pre‐, intra‐ and postoperative phases [3]. Within Enhanced Recovery After TBI (ERA‐TBI) pathways, core bundle elements include early mobilisation, structured multidisciplinary rehabilitation, nutritional and glycaemic optimisation, complication prevention and coordinated discharge planning [3]. Current evidence is strongest for early mobilisation and multidisciplinary rehabilitation strategies, while emerging interventions such as virtual reality (VR) and telerehabilitation remain investigational [3]. In elective craniotomy, recent systematic reviews and meta‐analyses report shorter hospital length of stay and improved pain and PONV with ERAS pathways, without clear increases in complications [4]. For broader context, cross‐speciality ERAS evidence shows reduced length of stay and perioperative complications [5]. These findings support the potential relevance of ERAS principles to recovery after TBI.

Despite standardised acute care, outcomes after TBI remain highly variable. Early, multidisciplinary rehabilitation is linked to shorter hospital stays and more efficient functional recovery without excess inpatient mortality [6]. At the system level, care‐pathway heterogeneity is pronounced across centres, with marked variation in outcomes even within high‐income regions [7]. Patient factors shape prognosis: advanced age remains a dominant predictor of higher mortality and unfavourable outcomes, and socioeconomic context influences discharge destination and length of stay [8]. Moreover, preexisting conditions including multimorbidity and metabolic disease are associated with higher complication rates and mortality, though findings are not uniform across studies [9, 10]. These inconsistencies and scattered comparisons highlight the need for a focused synthesis on recovery‐phase factors relevant to enhanced‐recovery TBI pathways.

This narrative review synthesises evidence on recovery‐phase factors that determine morbidity and mortality after TBI within enhanced‐recovery (ERA‐TBI) pathways. We focus on the recovery phase, specifically rehabilitation timing and intensity, and patient characteristics such as demographics and preexisting conditions. Perioperative/surgical details and acute ICU monitoring are outside scope, except where they contextualise recovery. After a short methods review, we examine three domains: rehabilitation approaches, patient demographics and preexisting conditions. We subsequently integrate findings in a discussion outlining implications and limitations for ERA‐TBI.

## 2. Methods

A targeted literature search was conducted using PubMed/MEDLINE and Google Scholar. Search terms combined MeSH terms and free‐text keywords, including: ‘Traumatic Brain Injury,’ ‘TBI,’ ‘Rehabilitation,’ ‘early mobilisation/early mobilisation,’ ‘multidisciplinary rehabilitation,’ ‘Enhanced Recovery After Surgery,’ ‘ERAS,’ ‘morbidity,’ ‘mortality,’ ‘functional recovery,’ ‘length of stay,’ ‘frailty,’ ‘socioeconomic status,’ ‘diabetes,’ ‘hyperglycaemia,’ ‘comorbidity’ and ‘risk stratification.’ Literature was searched from database inception.

Studies were included if they examined TBI populations and discussed recovery‐phase factors relevant to ERA‐TBI, including rehabilitation strategies, multidisciplinary care, patient demographics, socioeconomic status (SES), frailty or preexisting comorbidities. Eligible outcomes included morbidity, mortality, functional recovery, neurological recovery, quality of life, length of stay, complications, discharge destination and readmission.

Studies were excluded if they focused exclusively on non‐TBI populations, acute surgical technique, intraoperative care, anaesthetic protocols or ICU monitoring without recovery‐phase outcomes. Animal studies, laboratory‐only studies, editorials, commentaries, conference abstracts without full text, non‐English articles, duplicates and studies not directly relevant to ERA‐TBI recovery pathways were also excluded.

## 3. Rehabilitation Strategies

There are several rehabilitation strategies evidenced in the literature that can aid in reducing morbidity and mortality after TBI.

Incorporating early physical activity after TBI significantly increases neurological recovery, motor function and quality of life [11]. Patients who received early mobilisation therapy had a mean difference (MD) of −4.51 on the National Institutes of Health Stroke Scale (NIHSS), an MD of +5.39 on the Fugl–Meyer Assessment (FMA) and a standardised MD of 0.80 for quality of life [11]. When compared to early in bed upright position rehabilitation therapy, early out of bed mobilisation therapy is more effective as it leads to reduced ICU stays (by 5.9 days) and ventilation duration (by 6.7 days) [12].

Cognitive impairments can lead to reduced psychosocial activity in patients; thus, cognitive and neuropsychological interventions target language, perception, attention, memory and executive functioning. Cognitive therapy can be divided into two categories: restorative, which focuses on restoring impaired areas, and compensatory, which aims to discover methods to compensate for affected skills [13]. Neuropsychological assessment investigates how an individual’s thoughts, emotions and behaviour have been affected after TBI, to then create rehabilitation plans targeted to the patient’s specific weaknesses, goals and needs [14].

New technology such as VR, robotics, brain–computer interface and telerehabilitation are also proving to provide a unique environment for patients to recover [14]. When comparing VR‐based cognitive therapy with standard cognitive therapy, VR therapy had better outcomes: Montreal Cognitive Assessment score of 26.1 vs. 24.3, Frontal Assessment Battery of 16.35 vs. 15.15 and faster Trail Making Test of 111.9 vs. 226.3 s, respectively [15].

## 4. Patient Demographics

Patient demographics serve as foundational determinants of morbidity and mortality within the ERA‐TBI framework, often dictating the ‘biological reserve’ available for recovery. Advanced age is consistently cited as the most significant demographic predictor of poor outcomes [8]. Older patients experience higher rates of mortality and unfavourable functional recovery, largely due to age‐related brain atrophy and reduced neuroplasticity, which limit the brain’s ability to reorganise following trauma [16]. However, recent shifts in ERA‐TBI research emphasise “frailty” over chronological age, noting that physiological resilience often better predicts the ability to tolerate intensive rehabilitation [17].

SES also plays a critical role in the “morbidity” aspect of recovery. Patients from lower SES backgrounds often face disparities in discharge destinations; they are less likely to be transitioned to high‐intensity inpatient rehabilitation facilities and more likely to be discharged home with insufficient support [8, 18]. This “system‐level” demographic factor influences the length of stay and the likelihood of readmission. Within ERA‐TBI pathways, targeted interventions such as early social work involvement, discharge planning support and improved access to community rehabilitation resources may help mitigate these disparities and improve continuity of care following hospital discharge. Furthermore, while sex differences remain a subject of ongoing study, some data suggest that hormonal profiles may influence inflammatory responses post‐TBI, though clinical evidence regarding sex as a primary driver of mortality remains heterogeneous [19].

## 5. Preexisting Conditions

The presence of comorbidities significantly complicates the ERA‐TBI pathway, often acting as “silent” drivers of secondary brain injury. Metabolic health, particularly glucose regulation, is a primary concern. Preexisting diabetes mellitus and stress‐induced hyperglycaemia (even in non‐diabetics) are strongly associated with increased neuroinflammation and higher mortality rates [9, 20]. Within an enhanced recovery protocol, early glycaemic control is vital, as fluctuating blood sugar levels can exacerbate cerebral oedema and metabolic crisis in the penumbra of the injury [10].

Beyond metabolic factors, cardiovascular multimorbidity, such as preexisting hypertension or atrial fibrillation, increases the risk of perioperative complications, including secondary ischaemic strokes or venous thromboembolism (VTE) [10]. In addition, preexisting mental health conditions and substance use disorders (specifically alcohol and opioid use) are highly prevalent in the TBI population. These conditions are linked to ‘care‐pathway heterogeneity,’ as they can complicate delirium management, prolong hospital stays and increase the risk of postdischarge mortality [21]. Integrating these baseline health factors into a “prehabilitation” or “risk‐stratification” model is essential for the success of any ERA‐TBI protocol [22].

## 6. Discussion

ERA‐TBI protocols are multifactorial and are designed to reduce morbidity and mortality to ultimately improve patient outcomes. They are dependent on the type of rehabilitation strategy used, patient demographics and the presence of any preexisting conditions in patients. Older age and lower SES were the main patient factors that saw a rise in mortality and morbidity. Comorbidities such as diabetes mellitus, stress‐induced hyperglycaemia, hypertension, atrial fibrillation, mental health conditions and substance use disorders can contribute to cellular energy dysfunction in brain tissue, further complications (ischaemic strokes or VTE), longer hospital stays and postdischarge mortality. Therefore, creating a rehabilitation plan that takes into consideration all these factors, as well as patient needs, deficits and abilities, is crucial to achieving better patient health. Risk‐stratification within ERA‐TBI pathways may therefore benefit from incorporating frailty indices, multimorbidity assessments and early metabolic screening such as glucose dysregulation, which could help identify patients requiring intensified rehabilitation, closer monitoring or prolonged multidisciplinary support. Rehabilitation strategies that are commonly used or are emerging include early mobilisation, cognitive and/or neuropsychological therapy and the use of new technology such as VR. Educating and enabling patients to participate in effective rehabilitation methods can help reduce morbidity and mortality and increase quality of life, patient well‐being and independence.

A few notable limitations were observed when analysing the selected articles. Some studies had a small sample size of less than one hundred, reducing statistical power and the ability to draw strong conclusions. There is heterogeneity in the factors related to rehabilitation interventions, including the usage of pharmacotherapy in combination with different therapies, resource settings, patient population, severity of TBI and timing of implementation and outcome measurement. The lack of standardisation restricts the ability for direct comparison against multiple studies. The follow‐up period for the implementation of the rehabilitation therapies is often short, so although morbidity rate is seen to improve, mortality is not known or recorded and is therefore stated to be unaffected.

The subject of ERA post‐TBI and the factors determining mortality and morbidity is extensive, and there are many opportunities for future research in the area. There is currently a lack of research exploring the involvement of the multidisciplinary team in the rehabilitation programme. Within ERA‐TBI pathways, early multidisciplinary involvement within the first 48–72 h of recovery may include physiotherapists facilitating mobilisation and respiratory optimisation, occupational therapists assessing functional independence and discharge needs, speech and language therapists evaluating swallowing and communication deficits and psychologists or neuropsychologists identifying cognitive and behavioural impairments requiring targeted intervention. Roles such as occupational therapist, speech therapist, physiotherapist, psychologist and neurologist are all important to restore or improve motor, cognitive and emotional functioning as well as the seamless integration of patients into the community and daily living. The impact of more patient demographics on morbidity and mortality can be investigated so that stronger conclusions can be drawn. Examples include sex, genetics, ethnicity and geographical location. It would also be beneficial to look at the long‐term effects of the different rehabilitation interventions for a comprehensive view on their efficacy and impact. There are a limited number of studies on the use of innovative technology in rehabilitation, so exploring this area may be promising for the future of ERA‐TBI.

## 7. Conclusion

The implementation of ERA‐TBI pathways represents a necessary evolution from reactive acute care to a proactive, evidence‐based recovery model. This review highlights that while the primary injury is a fixed event, morbidity and mortality are significantly modulated by the interplay of clinical strategy and patient‐specific factors. Early, high‐intensity rehabilitation serves as the clinical engine of the ERA‐TBI protocol, leveraging neuroplasticity to reduce hospital length of stay and functional disability. However, the efficacy of these interventions is fundamentally constrained by patient demographics, where advanced age and socioeconomic barriers remain dominant predictors of unfavourable outcomes.

Furthermore, the “biological baseline” established by preexisting conditions—specifically metabolic and cardiovascular comorbidities—acts as a critical determinant of secondary injury severity. For ERA‐TBI pathways to be successful, they must move beyond “one‐size‐fits‐all” bundles and incorporate rigorous risk‐stratification that accounts for these baseline vulnerabilities. Future research should focus on refining the timing of “ultra‐early” mobilisation and developing targeted interventions for multimorbid patients. Ultimately, standardising the recovery phase through the ERA‐TBI framework offers the potential to bridge the gap between survival and functional independence, reducing the global burden of TBI‐related disability.

## Funding

The authors have nothing to report.

## Conflicts of Interest

The authors declare no conflicts of interest.

## Data Availability

The data that support the findings of this study are available from the corresponding author upon reasonable request.
